# Epibiotic pressure contributes to biofouling invader success

**DOI:** 10.1038/s41598-017-05470-2

**Published:** 2017-07-12

**Authors:** Kaeden Leonard, Chad L. Hewitt, Marnie L. Campbell, Carmen Primo, Steven D. Miller

**Affiliations:** 1 Institute for Marine and Antarctic Studies, University of Tasmania, Launceston, 7250 Tasmania Australia; 2 School of Science, University of Waikato, Hamilton, New Zealand; 3 Environmental Research Institute, University of Waikato, Hamilton, New Zealand; 4 School of Computing and Mathematical Sciences, University of Waikato, Hamilton, New Zealand

## Abstract

Reduced competition is a frequent explanation for the success of many introduced species. In benthic marine biofouling communities, space limitation leads to high rates of overgrowth competition. Some species can utilise other living organisms as substrate (epibiosis), proffering a competitive advantage for the epibiont. Additionally, some species can prevent or reduce epibiotic settlement on their surfaces and avoid being basibionts. To test whether epibiotic pressure differs between native and introduced species, we undertook *ex situ* experiments comparing bryozoan larval settlement to determine if introduced species demonstrate a greater propensity to settle as epibionts, and a reduced propensity to be basibionts, than native species. Here we report that introduced species opportunistically settle on any space (bare, native, or introduced), whereas native species exhibit a strong tendency to settle on and near other natives, but avoid settling on or near introduced basibionts. In addition, larvae of native species experience greater larval wastage (mortality) than introduced species, both in the presence and absence of living substrates. Introduced species’ ability to settle on natives as epibionts, and in turn avoid epibiosis as basibionts, combined with significantly enhanced native larval wastage, provides a comprehensive suite of competitive advantages contributing to the invasion success of these biofouling species.

## Introduction

Human mediated marine biological introductions have contributed to the increasing modification of global communities^1, 2^, with associated impacts to environmental, economic, social and cultural values^3^. Predicting successful invasions and the subsequent impacts and spread have remained central to invasion ecology^4–6^. Many new introductions experience a “release from enemies” in invaded regions due to the reduced probability of simultaneous introduction of both predator: prey or parasite: host pairs^7^, niche gaps in the invaded community^8^, or because of the naïveté of native enemies^9^.

Marine biofouling communities are often space-limited^10, 11^, typified by high competition through lateral overgrowth^10–12^, frequently resulting in mortality of the weakest competitor^11^. Yet many marine biofouling communities are heavily invaded^2, 13, 14^, with many introduced species obtaining spatial dominance^11–14^. Biofouling species represent a disproportionate number of total recognised invasions and impacts^1, 2^ resulting in global concern^15^. Entry into space limited systems requires new recruits to find suitable free substrate to settle and grow, however any free substrate can quickly become colonised during periods of high recruitment pressure^13, 16^. Settlement however is not restricted to inanimate substrates and frequently occurs on the living surfaces of marine biota. Epibiosis is the settlement and growth of one organism (epibiont) on another (basibiont)^17^. The majority of sessile and sedentary invertebrates and many algae are subject to epibiosis as a direct consequence of limited settlement substrate^18^.

Epibionts overcome the limitation of available substrate by exploiting other species as primary space. In addition, they also avoid edge contact that typically initiates competitive responses^17, 19^. Similar to recruitment onto bare space, individuals that live as epibionts exhibit preference when selecting a settlement site^20^. A high degree of co-evolution, adaption and specialisation between epibionts and basibionts has been hypothesised and described. This can be considered especially true with regards to the epibionts, as adequate settlement ensures success and survival^21^. Although a majority of epibionts do not restrict settlement exclusively to one host or exhibit obligate epibiosis^22^, some are considered to show strong selection preference based on properties of the basibiont^21, 22^. Moreover, analyses have shown that certain species that exhibit epibiosis also show topographic preference when settling on certain basibionts^23^, often choosing substrate characteristics suited to the epibiont’s requirements^24^.

Several researchers have shown positive outcomes of epibiosis for the basibiont, including protection^25^ and/or increased nutrients^21^. However, negative impacts are generally cited as outweighing benefits for many marine invertebrates^16–21^. The external surfaces of most sessile and sedentary species serve as a medium for exchange of numerous substances including nutrients, waste, defensive metabolites, gas, and release of spores or larvae^21^. Since epibionts create a new interface between the basibiont and its external environment, they can negatively impact all of the aforementioned properties^23^. For individual species, dealing with epibiosis generally involves trade-offs between tolerance and investment into defence, which utilises resources^21^. Defence mechanisms generally involve morphological adaptions (in the form of antifouling defence adaptions)^26^ or chemical antifouling^27^.

With this myriad of negative and positive gains being present in epibiont/basibiont associations, it is often assumed that there is some degree of co-evolution between epibiont and basibiont, yet few works have critically evaluated the influence of co-evolutionary history on this association^28^. By exposing native and introduced basibiont communities to native and introduced larvae as potential epibiont recruits, we examined the epibiotic pressures on native and non-native species and tested the role of evolutionary history on this interaction. We collected colonies of aspect dominant and reproductively active encrusting (forming a crust or hard coating over the substrate) and arborescent (forming a tree-like structure protruding from the substrate) bryozoans (native encrusting: *Celleporaria bispinata, Celleporaria foliata;* native arborescent: *Virididentula dentata;* introduced encrusting: *Watersipora subtorquata*, *Schizoporella unicornis, Cryptosula pallasiana;* introduced arborescent: *Bugula neritina*) from natural populations in northern Tasmania, Australia, placed them in 20 L aquaria maintained in a 14 °C climate-controlled room.

A subset of encrusting species’ colonies were fragmented into segments containing a growing edge and active feeding zooids, transplanted to glass slides and allowed 14 days post-transplant recovery to create potential basibionts. After two weeks, the remaining colonies of all seven species were induced to spawn and larvae were collected for immediate use in dose experiments as potential epibionts. Each encrusting species’ transplanted colonies (test basibionts) were exposed to each of the seven species’ larvae (as potential epibionts) in a pairwise fashion (e.g. *C. bispinata* test colony exposed to *W. subtorquata* larvae). The test basibiont colonies were individually placed surface-down in a 150 ml container of 0.2 µm filtered seawater and 25–30 larvae (potential epibionts) were inoculated. Containers were placed in the 14 °C climate-controlled room for 24 h prior to evaluation. After 24 h we evaluated larval settlement as: 1) settled as an epibiont; 2) settled adjacent to the basibiont colony; and 3) not settled (larval wastage).

## Results and Discussion

**Figure 1:**
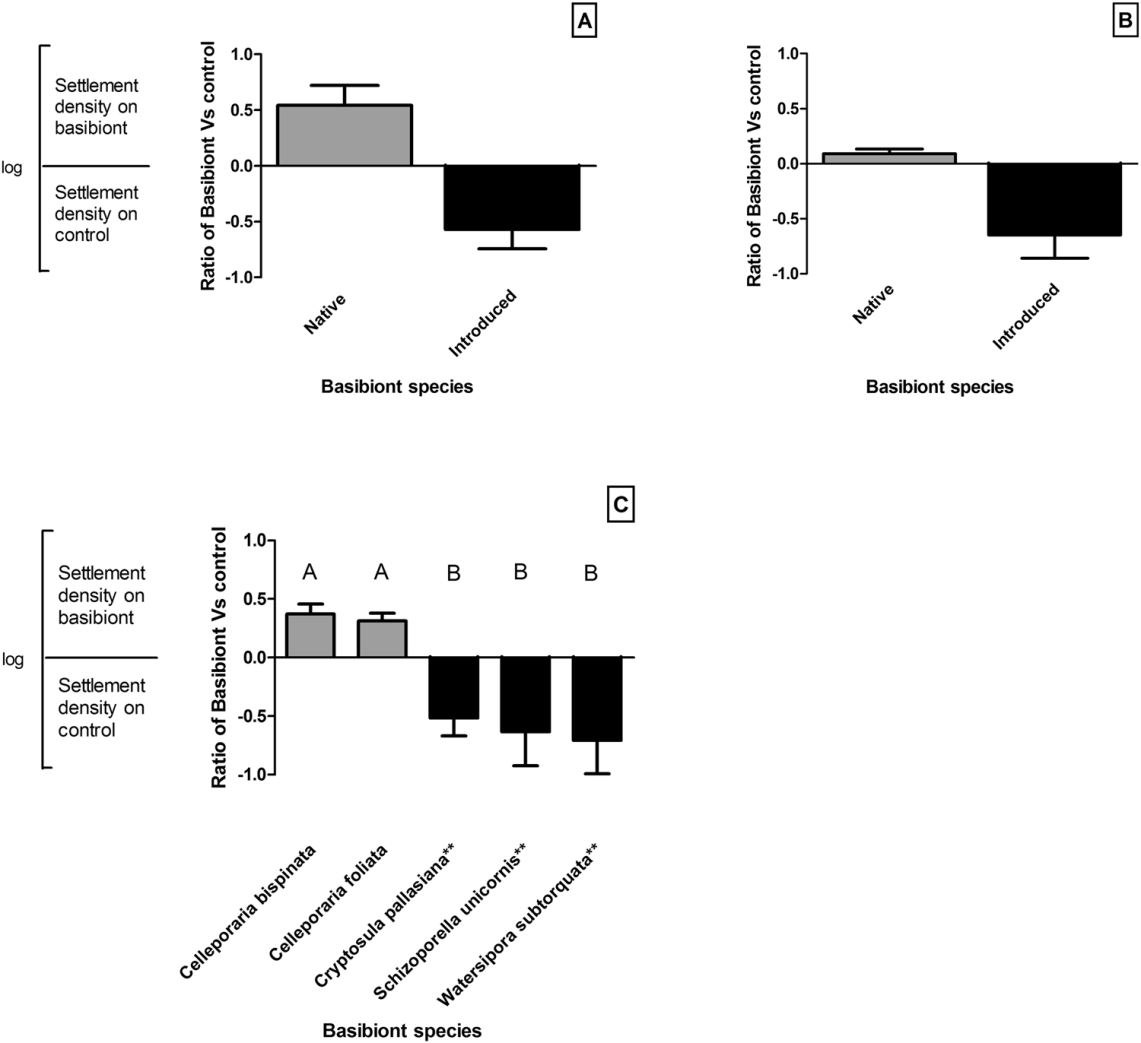
Epibiotic pressure on different basibiont species for: (**A**) native larvae; and (**B**) introduced larvae; (**C**) epibiotic pressure of combined native and introduced ratio settlement on different basibiont species (+SE, N = 70). Note: **Signifies introduced. Letters denote variations between test substrates.

Epibiotic settlement was generally low, with an average of 1.8% (±0.006 SE) and a maximum of 19.1% of available larvae settling as epibionts in any pairwise comparison. Native larvae settle as epibionts on native basibionts at greater densities than background settlement onto control bare space (*U* = 1.00, *P = *0.0008; Fig. 1A), and at higher levels on native basibionts than on introduced basibionts. Conversely, introduced larvae settle as epibionts on native basibionts at densities similar to control substrates and exhibited reduced settlement on introduced basibionts than control substrates (*U* = 29.50, *P = *0.0797; Fig. 1B). The total epibiotic pressure, that is the combined ratio of settlement of all epibionts (both native and introduced), compared to control substrates differed significantly (H_5_ = 19.53, *P < *0.0001; Fig. 1C). We found a very large effect where native basibiont colonies are 4 times more likely to have at least one epibiotic larval settlement than introduced basibionts (*U* = 34.0, *P = *0.0002; Hedges’ g = 1.497) and experience 10 times greater epibiotic density (individuals mm^−2^) by both native and introduced species than introduced basibionts (*U* = 166.0, *P = *0.0001; Hedges’ g = 0.807).

**Figure 2:**
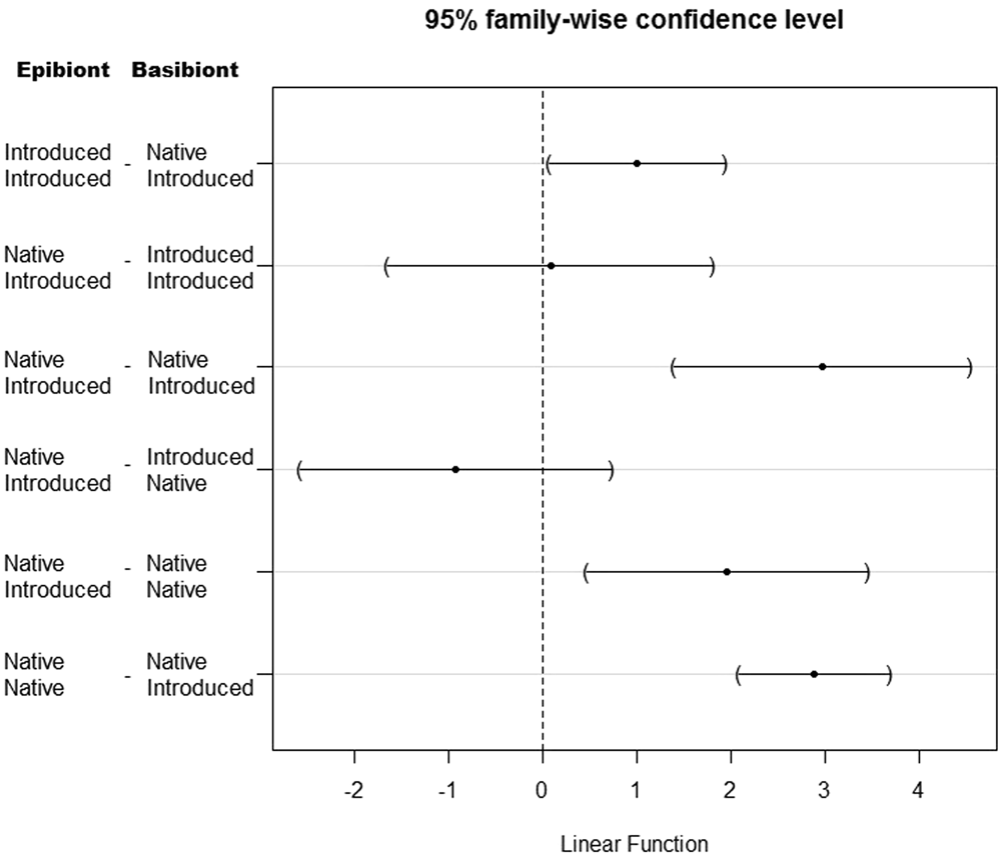
Plot of Tukey contrasts following a generalised mixed effects model comparing bryozoan larval settlement as epibionts on encrusting bryozoan basibionts.

The fit of a generalised mixed effects model (GLMM), allowing for an interaction between epibiont and basibiont species as a random effect, was not significantly different from a model without this interaction (χ^2^ = 1.679, df = 1, *P* = 0.195), therefore this interaction was omitted from the final model. Similarly the fixed effect of basibiont size was also found to not be significantly different (*Z* = 0.349, *P* = 0.727), resulting in its removal from the final model. The resulting (final) model found a significant interaction (*Z* = 0.485, *P* < 0.001) between epibiont status and basibiont status and the propensity for epibiont larvae to settle as epibionts on basibionts. Post hoc analysis revealed that larvae of introduced species have a significantly higher probability of settling as epibionts on native basibionts than on introduced basibionts (Fig. 2 and Table 1). Furthermore native epibionts have a significantly higher probability of settling on native basibionts than introduced epibionts settling on introduced basibionts, introduced epibionts settling on native basibionts, or native basibionts settling on introduced basibionts.

**Table 1:** Results of Tukey contrasts following a generalised mixed effects model comparing bryozoan larval settlement as epibionts on encrusting bryozoan basibionts.

Treatment	Comparison	Estimate	se	*Z* value	*P* value
	Epibiont + Basibiont − Epibiont + Basibiont				
On Test Substrates	Introduced + Native − Introduced + Introduced	1.00354	0.37343	2.687	**0.03109**
	Native + Introduced − Introduced + Introduced	0.08204	0.68712	0.119	0.99931
	Native + Native − Introduced + Introduced	2.96221	0.62641	4.729	**<0.00001**
	Native + Introduced − Introduced + Native	−0.92150	0.66018	−1.396	0.47053
	Native + Native − Introduced + Native	1.95867	0.59180	3.310	**0.00443**
	Native + Native − Native + Introduced	2.88017	0.31942	9.017	**<0.00001**

Early life history stages of many biofouling species are vulnerable to post-settlement mortality by competitors, and an ability to avoid intense competition has been hypothesised to influence larval site selection during settlement^13, 29^. Site suitability is assessed by physical and chemical cues, including the presence of extant community members. We tested whether biofouling larvae differentially responded to the presence of native versus introduced colonies by assessing proximal settlement response, evaluated as settlement adjacent to the test basibiont colonies, or environmental settlement response, measured as avoidance of settlement resulting in larval wastage (mortality).

**Figure 3:**
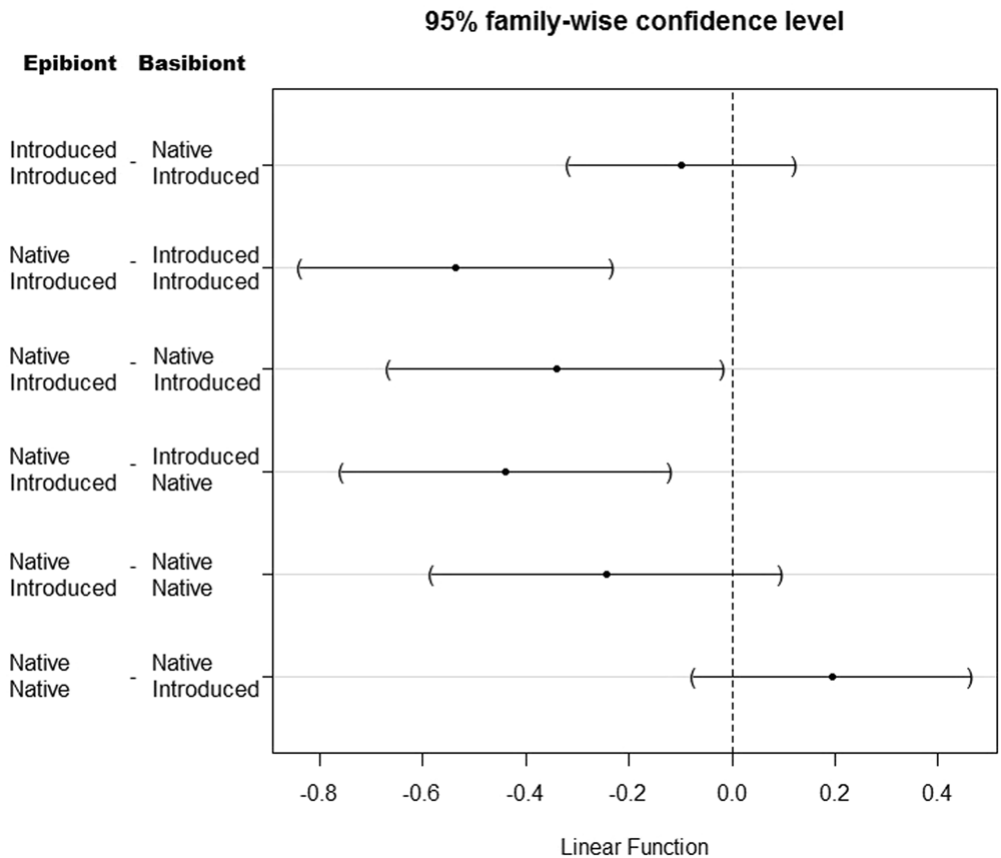
Plot of Tukey contrasts following a generalised mixed effects model comparing bryozoan larval settlement on space adjacent to test encrusting basibiont species.

A second full GLMM examined larval settlement on bare space adjacent to bryozoan test species, the random effect was found to be significantly different from a model without this interaction (χ^2^ = 10.602, df = 1, *P* < 0.0001), thus this effect was retained. However, again the size of the test basibiont colony was not significantly different (*Z* = 0.452, *P* = 0.6512), so was removed from the final model. The resulting model found a significant interaction (*Z* = 0.485, *P* < 0.001) between larval status (native/introduced) and their propensity to settle adjacent to native or introduced test basibiont species. The probability of introduced larvae settling adjacent to introduced test basibionts was significantly higher than native larvae settling adjacent to introduced test basibionts or native larvae settling adjacent to native test basibiont species (Fig. 3 and Table 2). Moreover introduced species are significantly more likely to settle adjacent to native basibiont species than native larvae are to introduced test basibiont species.

**Table 2:** Results of Tukey contrasts following a generalised mixed effects model comparing bryozoan larval settlement on space adjacent to test encrusting basibiont species.

Treatment	Comparison	Estimate	se	*Z* value	*P* value
	Epibiont + Basibiont − Epibiont + Basibiont				
Adjacent to Test Substrates	Introduced + Native − Introduced + Introduced	−0.09720	0.08593	−1.131	0.66112
	Native + Introduced − Introduced + Introduced	−0.53645	0.11851	−4.526	**<0.00001**
	Native + Native − Introduced + Introduced	−0.34165	0.12674	−2.696	**0.03366**
	Native + Introduced − Introduced + Native	−0.43925	0.12498	−3.515	**0.00241**
	Native + Native − Introduced + Native	−0.24445	0.13276	−1.841	0.24547
	Native + Native − Native + Introduced	0.19480	0.10502	1.855	0.23946

**Figure 4:**
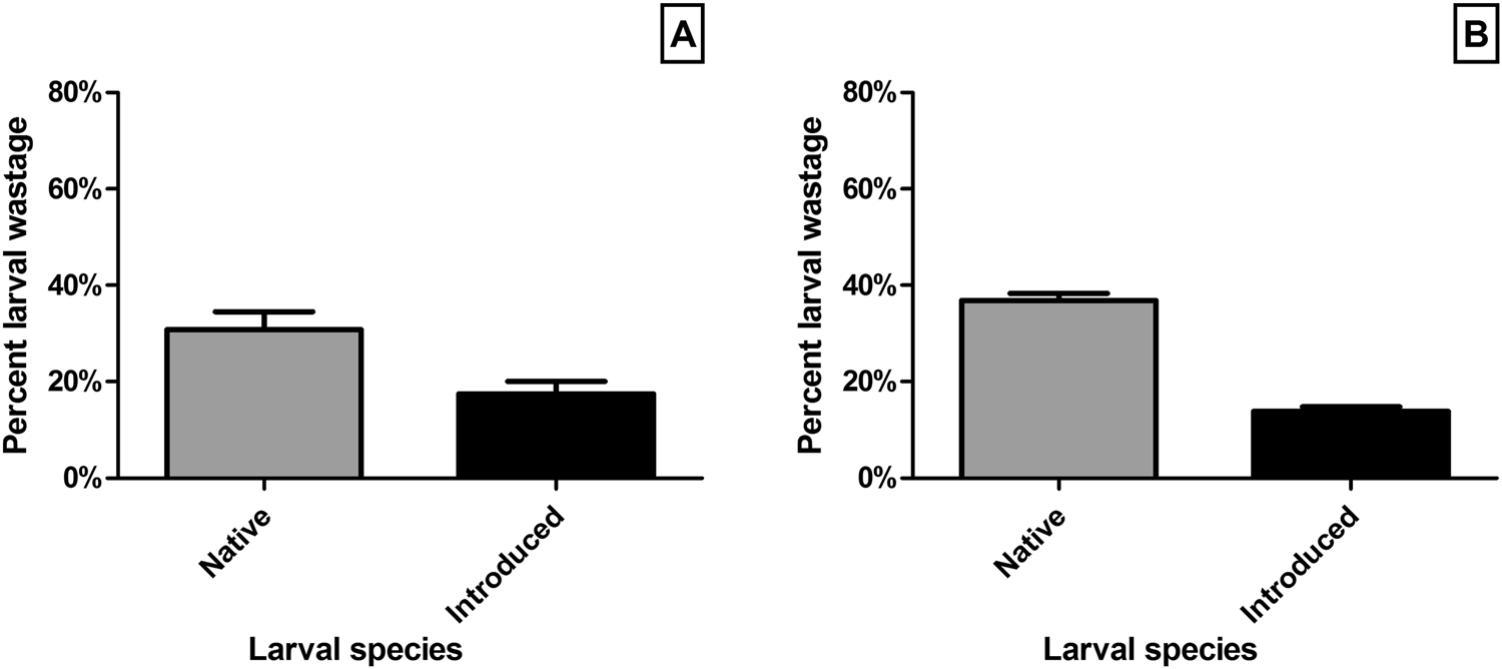
Larval wastage (non-settling larvae) for native and introduced larval (dose) species in the presence of (**A**) control and (**B**) test basibiont substrates (*C. foliata*; *C. bispinata*; *C. pallasiana*; *S. unicornis*; *W. subtorquata*) (±SE).

Native species were generally found to have a higher percentage (average 30.4% ±3.80%SE; n = 30) of larval wastage than introduced species (averaging 17.5% ±2.57%SE; n = 40) when exposed to control substrates alone – a medium effect (Hedges’ g = 0.698) of ~1.9 times greater larval wastage for natives (*U* = 368.5, *P* = 0.006). When exposed to the presence of basibionts, this differential effect becomes very large (Hedges’ g = 1.474) and increases to ~2.7 times, with native species exhibiting an average of 36.8% (±1.47%SE; n = 150) larval wastage and introduced species only 13.9% (±0.95%SE; n = 200) (*U* = 4403, *P* < 0.0001; Fig. 4).

Although the hypothesis that release from enemies may contribute to the success of introduced species in a new environment has received substantial support, previous focus has been on predators and parasites^7, 9^. Additionally, some studies have highlighted the role that competitive escape plays in enhancing growth and survival of some invaders^12, 13, 29^. Our results suggest that the insinuation of introduced species into biofouling communities is enhanced by a number of factors that create a feedback loop. Introduced species have the ability to settle on virtually any space (bare, native and introduced living substrate) thus avoiding competition and enhancing their survival, whereas the native species studied here are restricted in space for settlement (bare and native living substrate). As introduced species occupy more space, they have a large and differential effect on native larval choice thereby reducing the number of successful native settlers and enhancing native larval wastage but having limited effect on introduced settlers.

We note that the two native encrusting bryozoan species are congeners and therefore may not represent fully independent test subjects. While these findings may not be characteristic of all native bryozoan species, our results suggest important differences between native and introduced species’ settlement strategies and highlight ecologically important and previously under-appreciated effects of larval choice on invasion success in biofouling communities. More research is needed that includes non-phylogenetically related native bryozoans and in other locations to further explore if this pattern holds between different genera.

## Materials and Methods

### Study species and rearing

We selected bryozoans as the test phylum for this study because they form a hard substrate that is adequate for growth of other bryozoan species and exhibit both overgrowth competition and epibiotic relationships^10, 16, 17^. In addition, there is a suite of demonstrably native and introduced bryozoan species in Australia^30^. It is anticipated that there is an evolutionary response exhibited by basibionts in the form of defence, as the growth of an epibiotic bryozoan on the outside structure inhibits feeding and reduces gas exchange and potentially leads to the death of the bryozoan basibiont (*sensu* ^31^).

Colonies of reproductively mature and aspect dominant bryozoans were collected from the northern and eastern coasts of Tasmania, Australia, between November 2012 and February 2013 (Austral summer). Colonies collected comprised three native bryozoan species (encrusting: *Celleporaria bispinata* and *Celleporaria foliata;* and arborescent: *Virididentula dentata*) and four introduced bryozoan species (encrusting: *Watersipora subtorquata*, *Schizoporella unicornis* and *Cryptosula pallasiana;* and arborescent: *Bugula neritina*). We note that the two native encrusting bryozoan species are congeners and therefore may not be fully independent. Unfortunately none of the other native encrusting bryozoan species collected were also reproductively active.

Once removed from the water, all colonies were placed in separate containers, filled with seawater and placed in a cooler to narcotise the specimens and to reduce temperature fluctuations during transport to the laboratory at the University of Tasmania (UTAS) Launceston campus. Travel time between collection and delivery to the UTAS laboratory varied between 40 min to 2.5 h. On arrival at UTAS, colonies were placed into 20 L glass aquaria (W = 25 × L = 40 × H = 20 cm 20 cm) based on conspecific groups with 0.2 µm filtered seawater (FSW) and containing an air stone, in a climate controlled room held at 14 °C (equivalent to the ambient early spring water temperature). Bryozoan colonies were left to recover for at least 48 h before being used to create basibiont test substrates, or for inducing larval spawning.

## Experimental test substrates

### Control substrates

Seventy (ten for each of the seven inoculating species) 50 × 76 mm plain clear glass slides were maintained in 20 L aquaria with FSW and conditioned for 14 days as control settlement substrates, allowing a biofilm to establish^16^. FSW medium was changed every second day.

### Basibiont test substrates

The experimental design used 70 test substrates for each of the five experimental encrusting species (10 replicates for each of the seven inoculating species), each test substrate contained a living colony; arborescent colonies were not used as substrates. In the laboratory, colonies were removed from the substrate on which they were collected (primarily rock and shell) and fragmented into segments, resulting in various sizes ranging from ~59 mm^2^ to ~496 mm^2^, with a mean size of 246 mm^2^ (analysed with the software ImageJ^32^). Linear regression established that sizes of individual colonies (mm^2^) does not statistically predict rates of epibiotic settlement (counts of epibiosis) on any of the test basibiont species at *P* < 0.05 (Supplementary Figures S3 and S4).

Each segment of a colony included a growing edge of zooids and active feeding zooids. Segments were then transplanted onto a 50 × 76 mm plain clear glass slides using a non-toxic cyanoacrylate glue (Ecotech marine LLC Coral Glue). Care was taken to ensure that only a small amount of glue came in contact with the bottom of the colony and that the glue did not come in contact with the colony growing edge.

Individual slides were placed vertically into racks ensuring that faecal matter fell away from the colony^33^. Slides were returned to aquaria with FSW and left to recover and grow onto the glass substrate for 2 weeks before being used in settlement assays. Colonies were fed on *Rhodomonas baltica* once daily to a total aquaria concentration of approximately 100 cell µl^−1^; FSW was changed every second day^34^.

### Spawning and experimental inoculations

Larvae from brooding adult colonies of all of the seven species (five encrusting and two arborescent species) were used to assess epibiotic settlement preference (herein referred to as test larvae). The release of larvae from brooding adult colonies was induced as a response to light shock^35^. Colonies were placed into blacked out aquaria containing FSW and were fitted with an air stone to provide water movement. Bryozoan colonies were kept in the dark and left unhindered for 24–48 h to adapt to the experimental conditions. After this time, bryozoan colonies were placed in separate beakers with FSW and subjected to a bright ultraviolet (UV) light until spawning occurred. Typically, spawning began between 15 min and 1 h after exposure to light.

Initially larvae showed a positive phototactic response and were thus easily collected using a smaller, more directed, light source to attract them and a disposable 2 ml pipette. Larvae were collected within 10 min of spawning and immediately used to inoculate containers with the experimental test substrates (control or basibiont colonies).

### Experimental set-up

For the experiment, each individual slide containing an experimental test substrate was placed into a small container with 150 ml of FSW with the test substrate colony facing down. Tapered containers ensured the experimental colony was held at approximately 2 cm from the bottom of the container. Each container was inoculated with 25 to 30 spawned larvae. After inoculation, the containers were carefully moved back into the climate controlled room and maintained at a temperature of 14 °C +/− 2 °C for 24 h. Lights remained on within the room, but containers were shaded to best mimic cryptic environments.

Slides were examined after 24 h, noting the location of the larvae, where locations included: 1) settled as an epibiont; 2) settled on the glass slide adjacent to the basibiont; 3) settled within the container but not adjacent to or on the basibiont; and 4) not settled (larval wastage). Larvae were considered to be settled if they were attached and had metamorphosed.

### Data analysis

Differences between settlement of native and introduced larvae settling (as epibionts), or being settled on (as basibionts) compared to control substrates were examined to determine *epibiotic pressure* for an individual basibiont species. Ratio settlement was calculated by taking an individual species’ mean recruitment onto basibionts (individuals mm^−2^) and dividing it by the same species’ mean recruitment (individuals mm^−2^) onto control substrates. This standardised the analyses taking into account the size of the basibiont colony and specific differences in species’ larval survival. Ratios were log transformed to aid in the visual presentation of figures only, so as that greater settlement on basibionts is presented above the x-axis and greater settlement onto bare space is presented under the x-axis. Differences in settlement of native and introduced larval settlement onto unoccupied space adjacent to the basibiont test substrate (i.e., any bare space not occupied by a test species on a test substrate; hereon *adjacent ratio*) were evaluated in similar fashion.

Non-parametric tests were selected for data analysis because the data failed tests for normality, with data transformation failing to address this. Kruskal-Wallis analyses were used to evaluate differences when more than 2 groups were compared. Subsequently, pairwise comparisons were performed using Dunn’s procedure. This post hoc analysis is presented on figures with different letters marking significance; bars with corresponding letters were not significantly different. A Mann-Whitney U test was used only when two groups were compared. Hedges’ g was calculated to provide a measure of the effect size between native and introduced groups for epibiotic settlement and larval wastage. Hedges’ g is preferred for low sample sizes (n < 50), however the correction based on the pooled standard deviations was used.

A generalised mixed effects model (GLMM) with a binomial distribution and logit link function was used to analyse the differential behaviours of native and introduced species as epibionts or as basibionts. Basibiont and epibiont status was set as a full interaction fixed factor and individual species of epibiont and basibiont being modelled as crossed random effect factors. Additionally the model corrected for basibiont size and number of inoculated larvae.

Differences in settlement of native and introduced larval settlement onto unoccupied space adjacent to the basibiont test substrate (i.e., any bare space not occupied by a test species on a test substrate) were evaluated in similar fashion using a second GLMM with a binomial distribution and logit link function with the number of larvae settling on bare space adjacent to test basibionts as the dependant variable. Again we included test basibiont and larvae status as a full interaction fixed factor and individual species of larvae and test basibiont as crossed random effect factors, correcting for test basibiont size and number of inoculated larvae.

GLMM statistical analyses were performed using “R” (version 3.3.2)^36^, we implemented GLMM using the “lmer” function in the “lme4” package^37^. For post hoc analyses (Tukey tests) we used with the “glht” function of the “multcomp” package with p-values adjusted for multiple comparisons^38^.

## Electronic supplementary material

Supplementary Information
