# Supplementary material for: Epibiotic pressure contributes to biofouling invader success
Source: Sci Rep. 2017 Jul 12;7:5173. doi: 10.1038/s41598-017-05470-2 (PMC5507970; doi:10.1038/s41598-017-05470-2)
Supplement: Supplementary file 1 — Supplementary Information [file 41598_2017_5470_MOESM1_ESM.pdf]

Supplementary Information

Epibiotic pressure contributes to biofouling invader success

Kaeden Leonard, Chad L. Hewitt, Marnie L. Campbell, Carmen Primo and Steven D. Miller

Supplementary Information

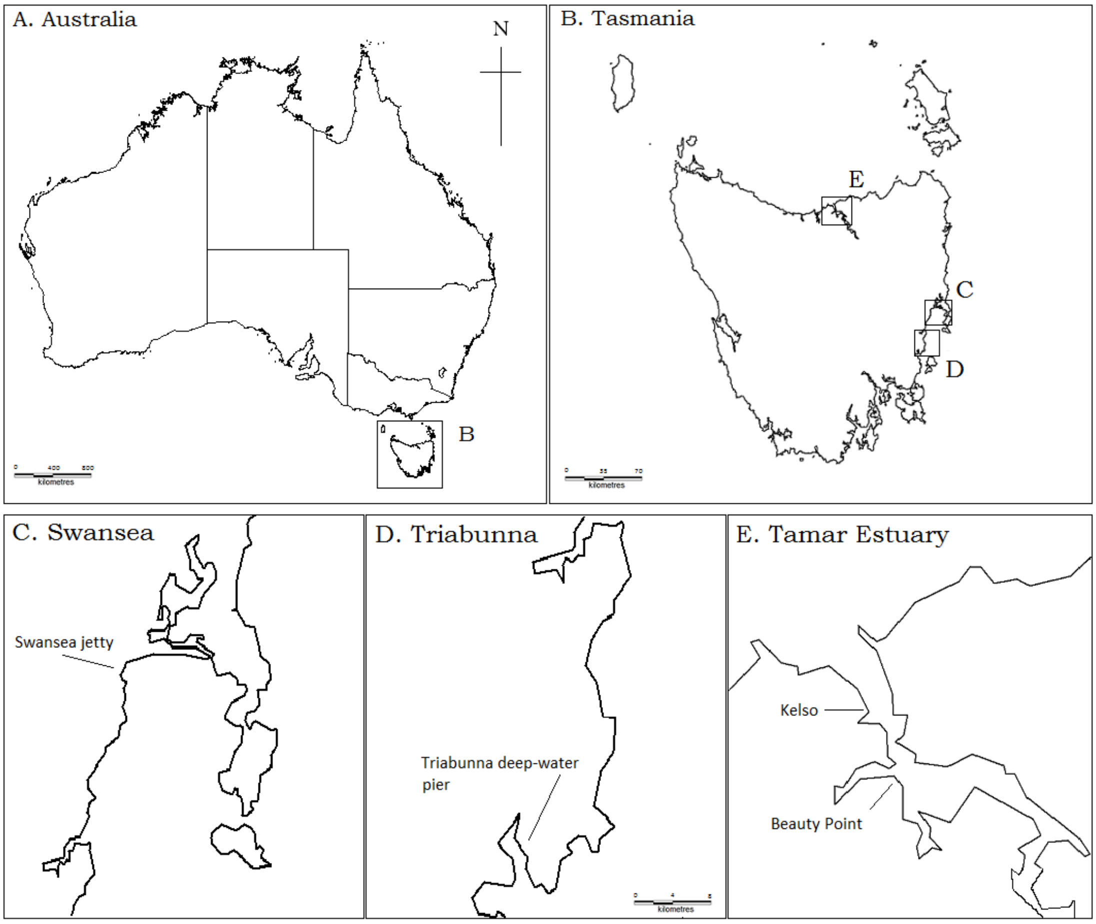

Figure S1: Tasmanian sample locations where bryozoan colonies were collected. Maps were generated in MapInfo pro 16.0, using maps obtained from © Commonwealth of Australia (Geoscience Australia) 2016. This product is released under the Creative Commons Attribution 4.0 International Licence. http://creativecommons.org/licenses/by/4.0/legalcode.

Table S1: Bryozoan colony collection information.

Species	Status	Collection location	Collection depth	Collection substrate
Celleporaria bispinata	native	Kelso, Tasmania (41.107°S, 146.799°E)	7m	PVC settlement panel
Celleporaria foliata	native	Beauty Point, Tasmania (41.157°S, 146.824°E)	5m	wooden wharf piles
Virididentula dentata	native	Kelso, Tasmania (41.107°S, 146.799°E)	5-7m	small rock and shell
Bugula neritina	introduced	Triabunna, Tasmania (42.520°S, 147.918°E)	<5m	wooden wharf piles
Cryptosula pallasiana	introduced	Beauty Point, Tasmania (41.157°S, 146.824°E)	2m	PVC settlement panel
Schizoporella unicornis	introduced	Triabunna, Tasmania (42.520°S, 147.918°E)	<5m	mussel shells
Watersipora subtorquata	introduced	Swansea, Tasmania (42.123°S, 148.078°E)	2m	wooden wharf piles

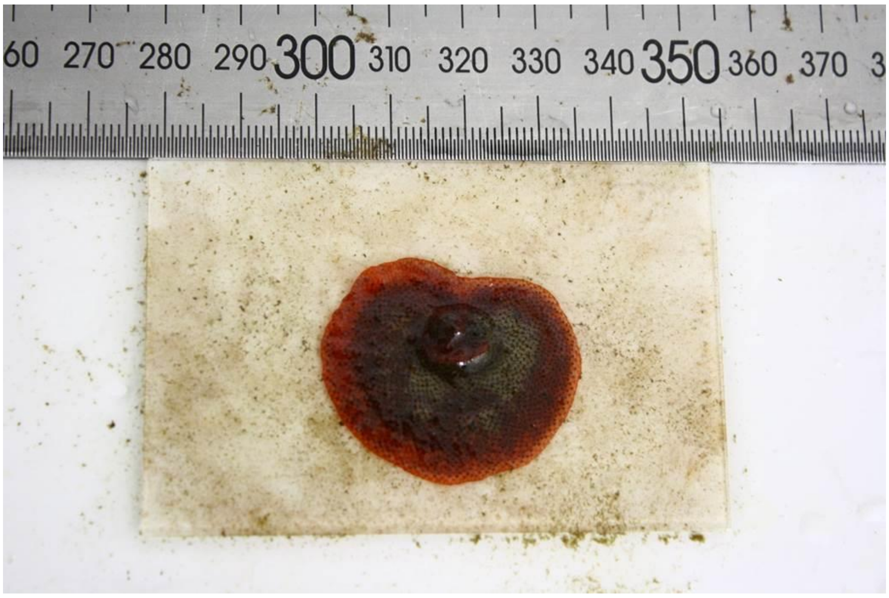

Figure S2: Example of basibiont test substrate on glass slide (*Watersipora subtorquata)* following 2 week post-transplant recovery.

Table S2: Basibiont test substrate sizes.

Test substrate species	Minimum	Median	Maximum	Mean	Std. Deviation	Std. Error
Celleporaria bispinata	59.46	208.6	383.8	209.7	80.22	9.589
Celleporaria foliata	61.79	211.5	471.9	224.1	88.97	10.63
Watersipora subtorquata	68.87	188.7	399.1	217.7	91.59	10.95
Cryptosula pallasiana	60.83	239.8	399.9	247.2	87.66	10.48
Schizoporella unicornis	121.9	312.7	495.7	329.9	82.85	9.903

Linear regression established that sizes of individual colonies (mm^2) could not statistically predict rates of epibiotic settlement (counts on individual basibionts) of native larval (Figure S3) or introduced larval (Figure S4) bryozoan species, on any of the test basibiont species at $p < .05$.

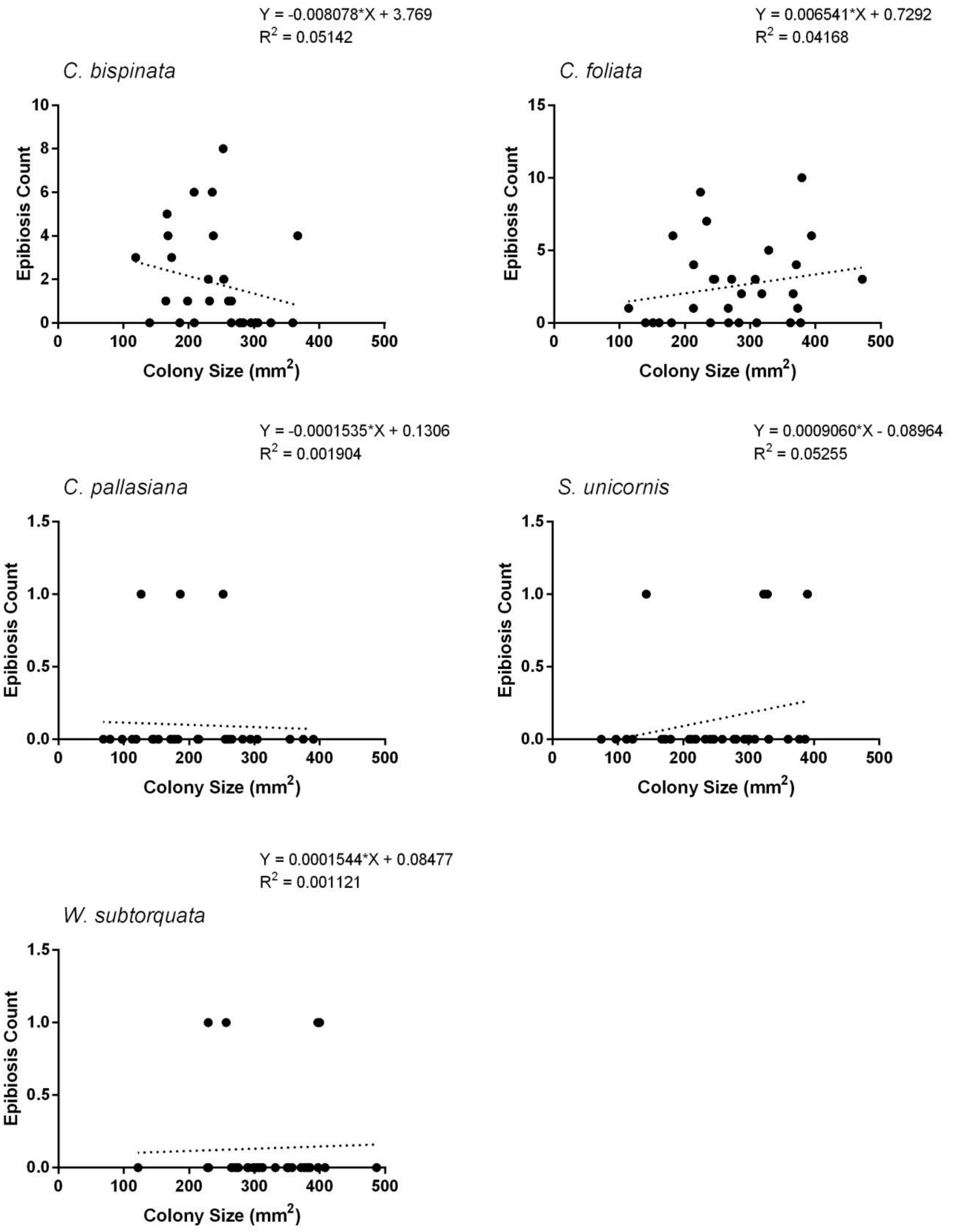

Figure S3: Scatter plots illustrating the relationship between counts of native bryozoan epibiotic settlement on test basibiont species versus colony size (mm^2). Note: y-axis ranges differ between graphs.

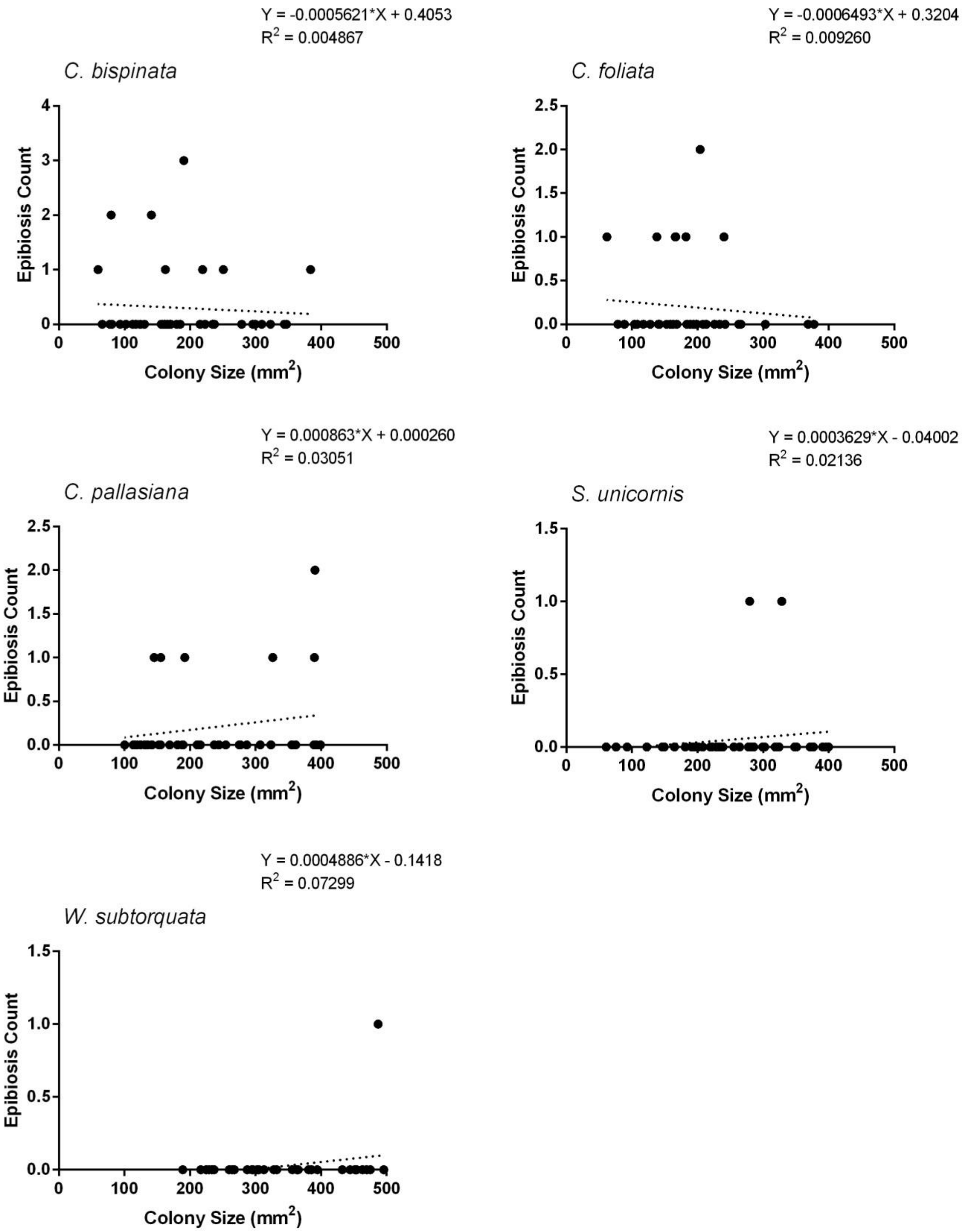

Figure S4: Scatter plots illustrating the relationship between counts of introduced bryozoan settlement on test basibiont species versus colony size (mm^2). Note: y-axis ranges differ between graphs.

Epibiotic settlement

Larval settlement onto living space as epibionts represented a small percentage of replicate basibiont treatments resulting in at least one epibiotic settlement (Figure S5A), and a low percentage of available larvae settling as epibionts in any pairwise comparison (Figure S5B). Native larvae settling on native basibionts were the exception to this general pattern with *C. bispinata* and *C. foliata* larvae settling as epibionts on more than 70% of *C. bispinata* and *C. foliate* basibiont test substrates and with greater proportions of total larvae (>6%) settling as epibionts, more than 3 times the percentage of introduced larvae and the native *V. dentata.*

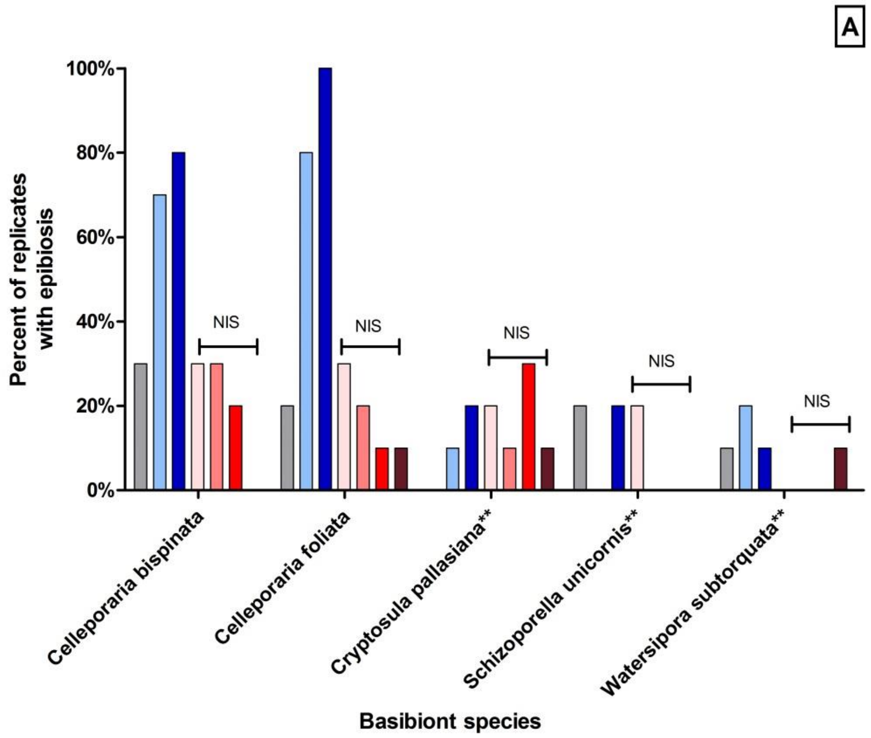

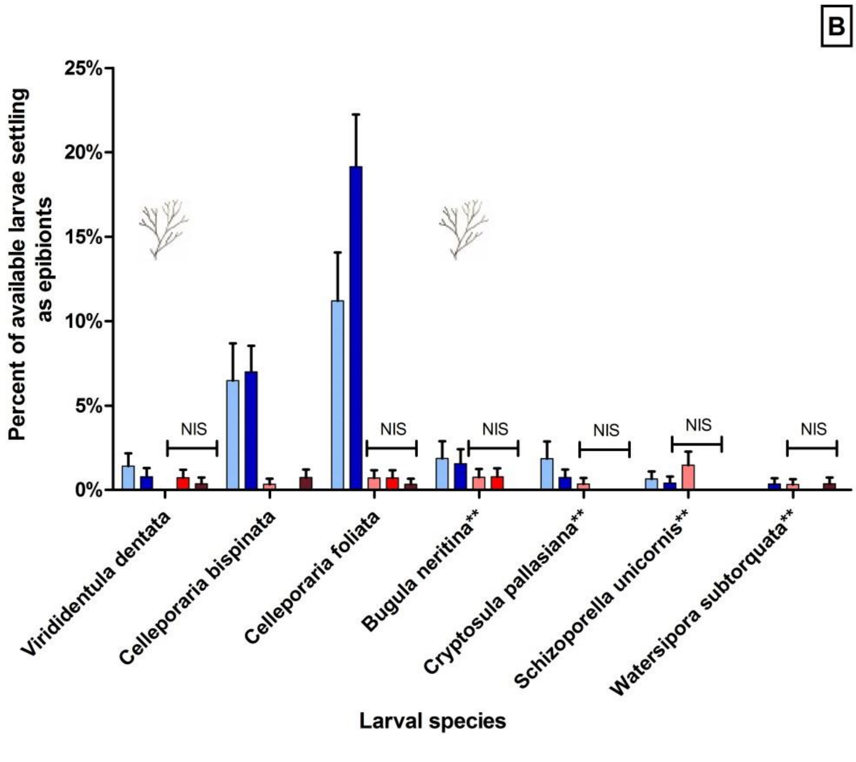

Figure S5: A) Percent of experimental replicates with epibiosis; B) Percent of larvae settling as epibionts on each basibiont species. *V. dentata* (grey); *C. foliata* (light blue); *C. bispinata* (dark blue); *B. neritina* (light pink); *C. pallasiana* (dark pink); *S. unicornis* (red); *W. subtorquata* (burgundy). Image over group indicates arborescent bryozoan species. (±SE, N=10). Note: ** and NIS (non-indigenous species) denotes introduced species.

Settlement adjacent to test basibiont substrates

To assess the influence of colony presence on larval settlement, we assessed the settlement densities on bare space immediately adjacent (i.e., any bare space not occupied by a test species on a test substrate) to the basibiont test substrates (individuals mm^{-2}) and compared densities with control substrates using log ratios in a similar fashion to the epibiosis analysis. Larval settlement onto space immediately adjacent to the test basibiont colonies was common; >99% of replicate basibiont treatments resulted in at least one epibiotic settlement, with average percent of larvae settling adjacent to test basibionts ranged between 20% and 55% of available larvae (Figure S6). Native larvae settled adjacent to basibionts at significantly lower densities than introduced species (t_{348} =10.93; P<0.0001), with 27% (on average) of native larvae settling adjacent to basibionts (native 28.8%; introduced 25.7%; t_{148} =1.556; P=0.121) whereas 41% of introduced larvae (on average) settled adjacent to basibionts (native 38.9%; introduced 43.5%; t_{198} =2.518; P=0.126).

The two arborescent bryozoans, *V. dentata* (native) and *B. neritina* (introduced), showed a preference to settle on adjacent bare space over basibionts (Figures S5 and S6), however exhibited a much higher preference to settle adjacent to basibionts test substrates over control substrates (Figure S5). In contrast, the two native encrusting bryozoans, *C. bispinata* and *C. foliata*, consistently settled at lower percentages than control substrates (Figure S6).

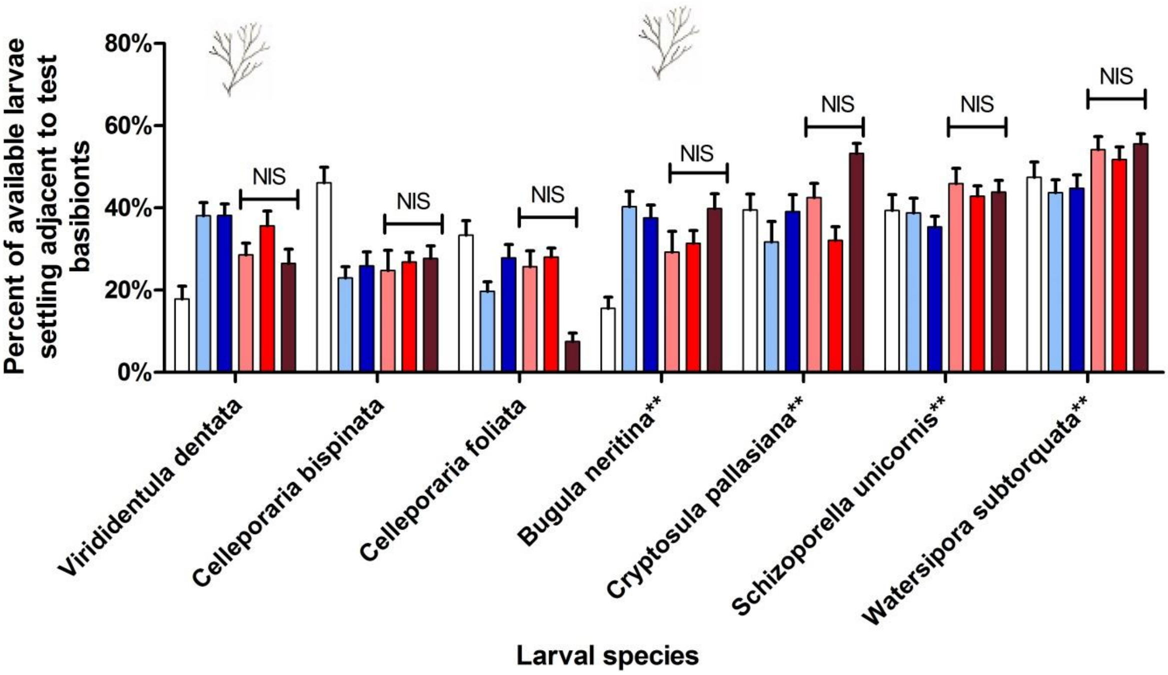

Figure S6: Percent of dosed larvae settling adjacent to basibionts. Control (white); *C. foliata* (light blue); *C. bispinata* (dark blue); *C. pallasiana* (dark pink); *S. unicornis* (red); *W. subtorquata* (burgundy). Image over group indicates arborescent bryozoan species. (±SE, N=10). Note: ** and NIS (non-indigenous species) denotes introduced species.

Larval wastage (non-settlement)

There was a significant difference in the percentage of larval wastage, measured as non-settlement on any substrate after 24 h, between different bryozoan species when inoculated onto bare control substrate (H_5=54.94, *P*<0.0001; Figure S7). Larval mortality of both *W. subtorquata* and *S. unicornis* was significantly lower than all other species apart from *V. dentata* and each other. Additionally, *V. dentata* had significantly lower larval mortality than *C. bispinata.*

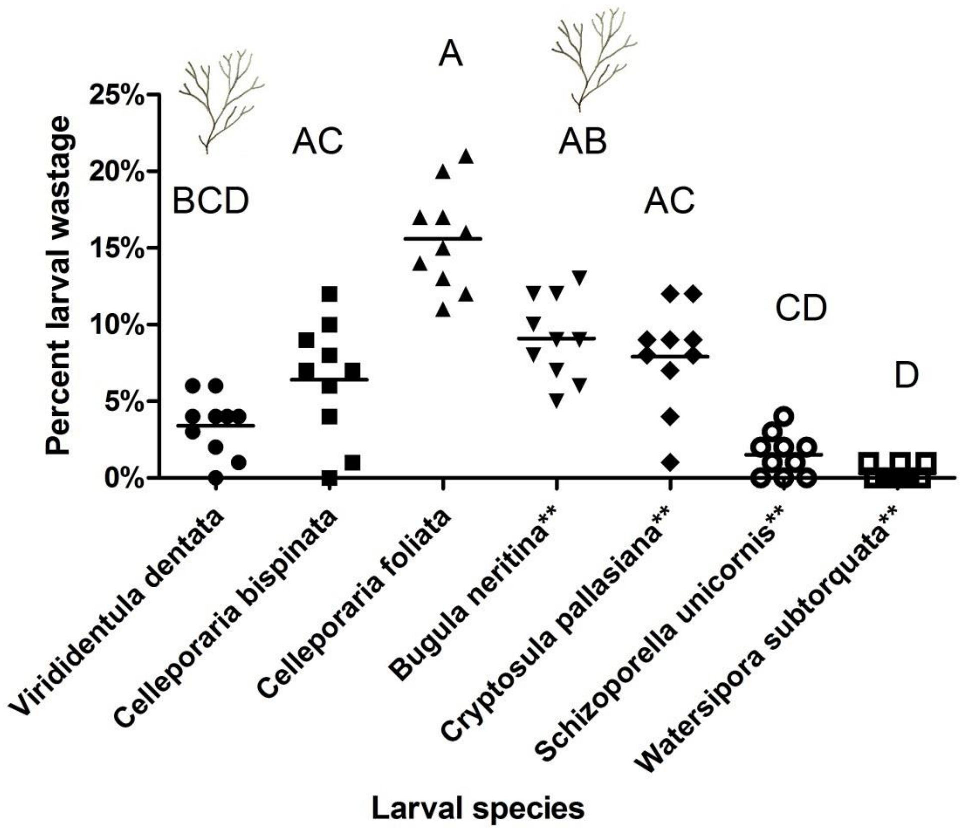

Figure S7: Percent larval wastage (non-settling larvae after 24h) when exposed to bare control substrate. Different data points represent mortality for each replicate; horizontal line represents mean settlement. Image over group indicates arborescent bryozoan species. Note: ** denotes introduced species.

A generalised mixed effects model (GLMM) was used to analyse differential larval mortality of native and NIS bryozoan species when settlement surfaces provided contained a native or NIS test basibiont colony. The model allowing for an interaction between epibiont and basibiont species as a random effect was not significantly different from a model without this interaction (χ^2 = 5.4244, df = 1, *P* = 0.01986), thus this interaction was retained, however, the fixed effect of basibiont size was not significantly different (*Z* = -0.397, *P* = 0.69146), so it was also removed from the model. The resulting model found a significant interaction (*Z* =-2.100, *P* < 0.03571) between larvae status and the level of mortality when test substrate contained a native or NIS colony. Post Hoc analysis showed that the probability of increased mortality was higher for native larvae when a NIS test basibiont was present than larvae of NIS when a NIS basibiont was present.

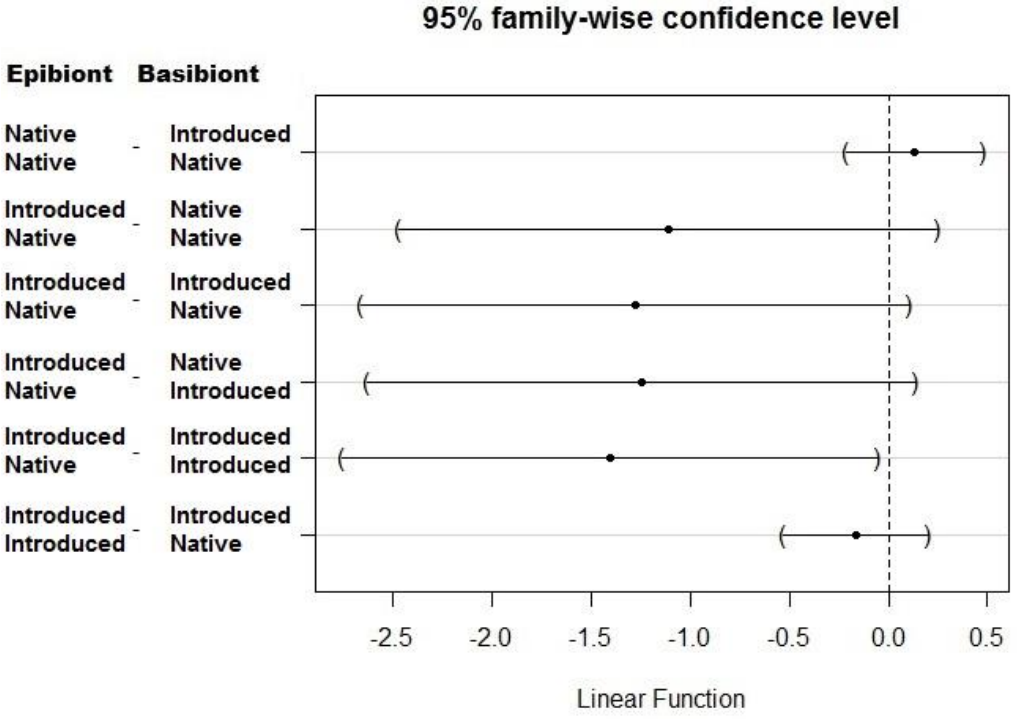

Figure S8: Plot of Tukey contrasts following a generalised mixed effects model comparing larval wastage (non-settling larvae after 24hr) on individual basibiont test substrates.

Table 3: Results of of Tukey contrasts following a generalised mixed effects model comparing larval wastage (non-settling larvae after 24hr) on individual basibiont test substrates.

**Comparison**<br>Epibiont + Basibiont - Epibiont + Basibiont	**Estimate**	**se**	***Z* value**	***P* value**
Native + NIS – Native + Native	0.1323	0.1423	0.930	0.7280
NIS + Native – Native + Native	1.1096	0.5583	-1.987	0.1459
NIS + NIS – Native + Native	-1.2735	0.5671	-2.246	0.0814
NIS + Native – Native + NIS	-1.2419	0.5670	-2.191	0.0926
NIS + NIS – Native + NIS	-1.4058	0.5550	-2.533	0.0384
NIS + NIS – NIS + Native	-0.1639	0.1514	-1.082	0.6274
